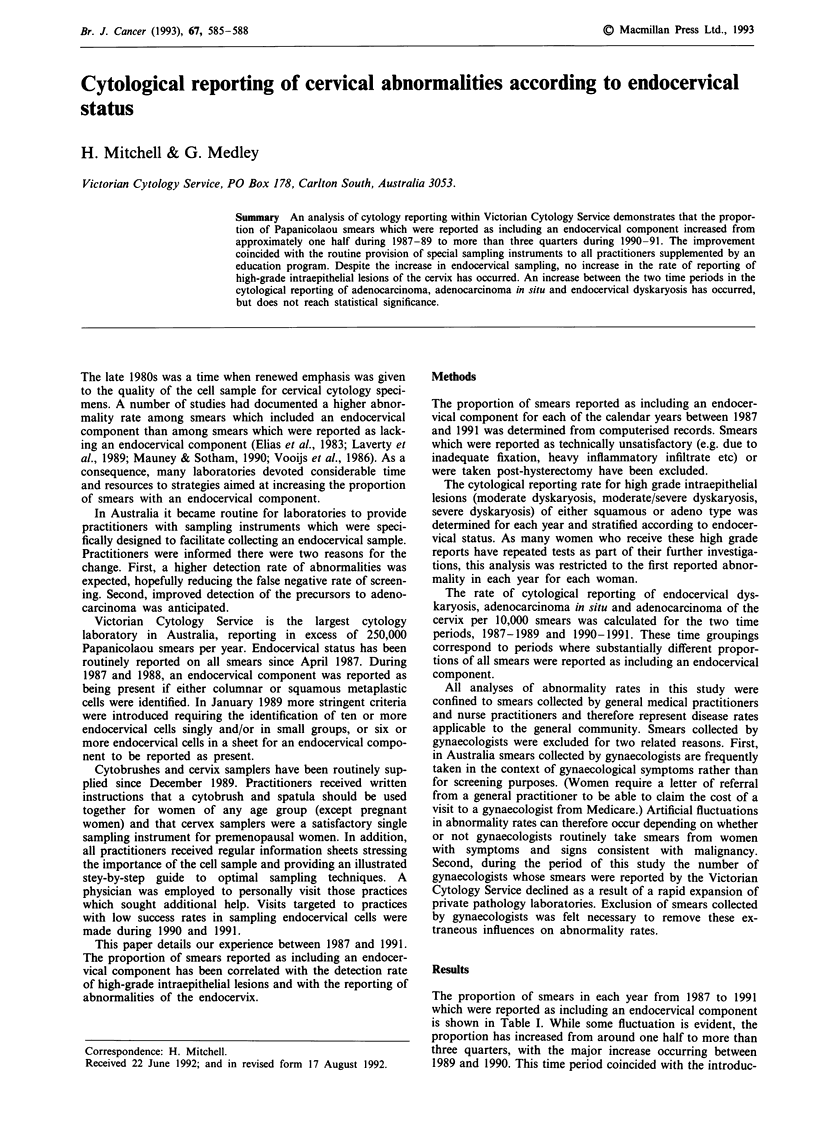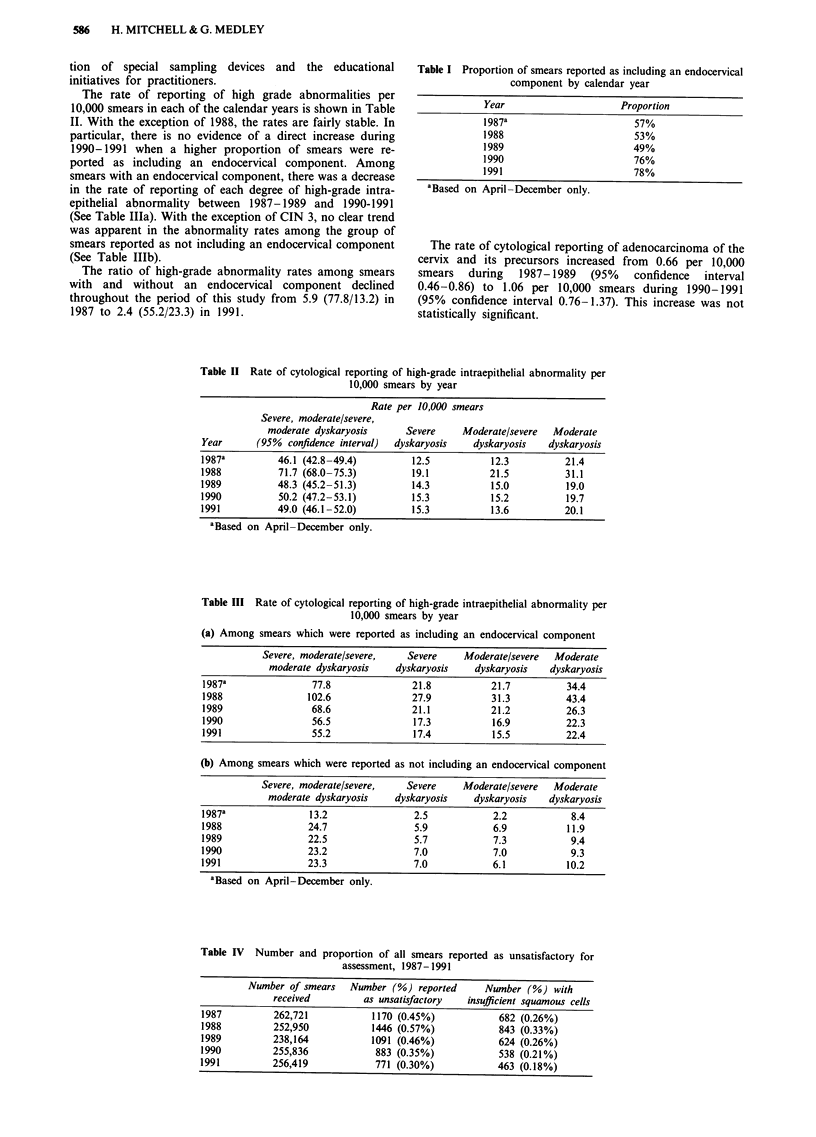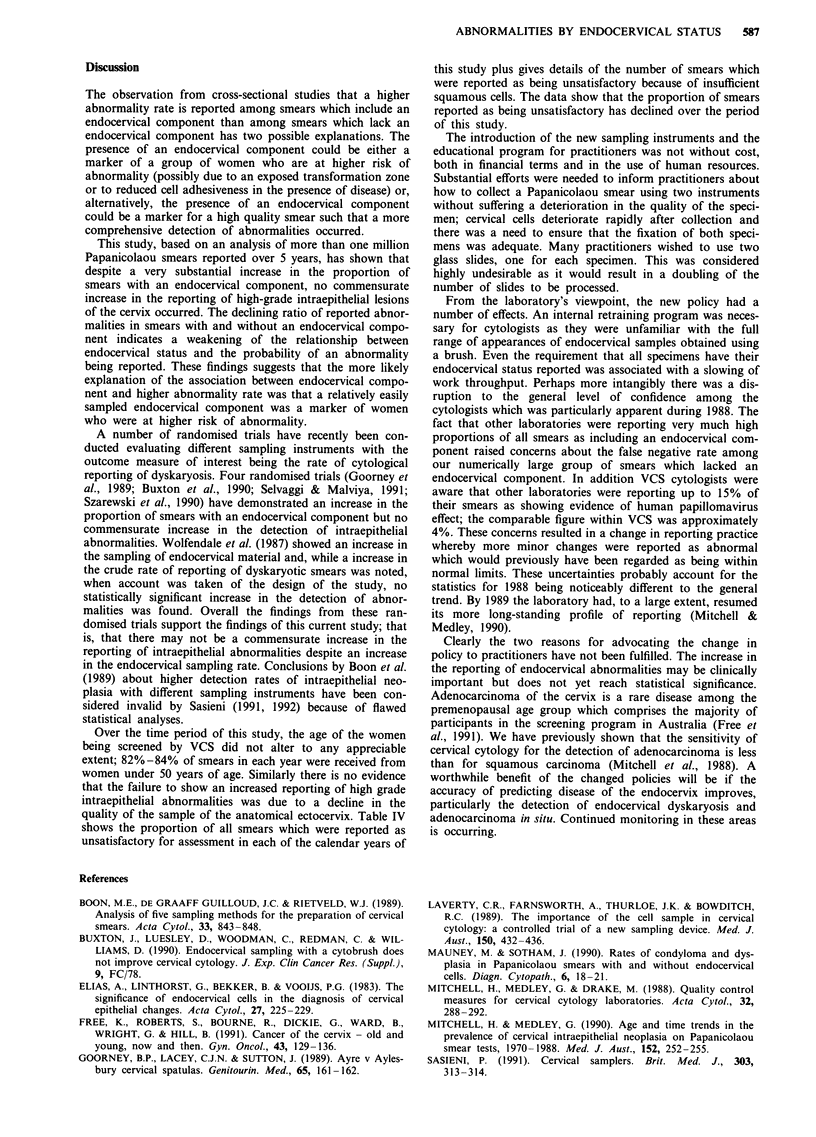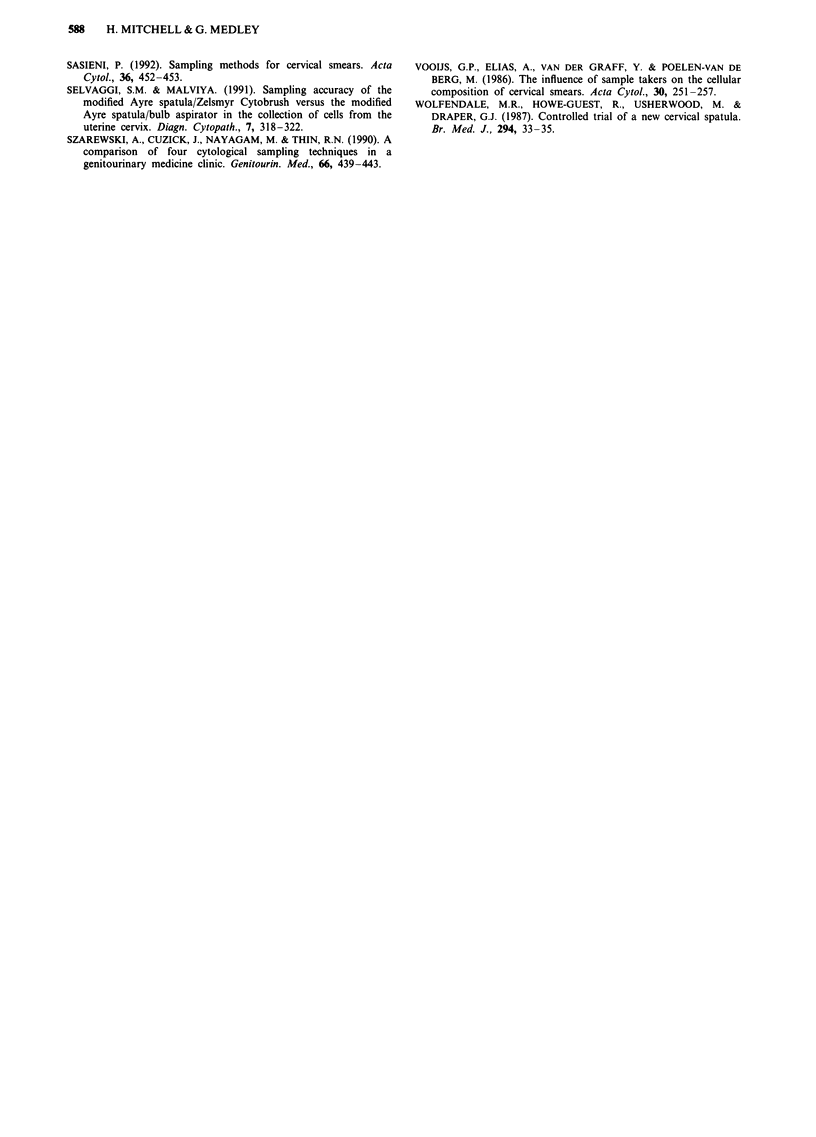# Cytological reporting of cervical abnormalities according to endocervical status.

**DOI:** 10.1038/bjc.1993.107

**Published:** 1993-03

**Authors:** H. Mitchell, G. Medley

**Affiliations:** Victorian Cytology Service, Carlton South, Australia.

## Abstract

An analysis of cytology reporting within Victorian Cytology Service demonstrates that the proportion of Papanicolaou smears which were reported as including an endocervical component increased from approximately one half during 1987-89 to more than three quarters during 1990-91. The improvement coincided with the routine provision of special sampling instruments to all practitioners supplemented by an education program. Despite the increase in endocervical sampling, no increase in the rate of reporting of high-grade intraepithelial lesions of the cervix has occurred. An increase between the two time periods in the cytological reporting of adenocarcinoma, adenocarcinoma in situ and endocervical dyskaryosis has occurred, but does not reach statistical significance.


Br. J. Cancer (1993), 67, 585-588 © Macmillan Press Ltd., 1993

### Cytological reporting of cervical abnormalities according to endocervical status

H. Mitchell & G. Medley

*Victorian Cytology Service, PO Box 178, Carlton South, Australia 3053.*

**Summary** An analysis of cytology reporting within Victorian Cytology Service demonstrates that the proportion of Papanicolaou smears which were reported as including an endocervical component increased from approximately one half during 1987-89 to more than three quarters during 1990-91. The improvement coincided with the routine provision of special sampling instruments to all practitioners supplemented by an education program. Despite the increase in endocervical sampling, no increase in the rate of reporting of high-grade intraepithelial lesions of the cervix has occurred. An increase between the two time periods in the cytological reporting of adenocarcinoma, adenocarcinoma *in situ* and endocervical dyskaryosis has occurred, but does not reach statistical significance.

The late 1980s was a time when renewed emphasis was given to the quality of the cell sample for cervical cytology specimens. A number of studies had documented a higher abnormality rate among smears which included an endocervical component than among smears which were reported as lacking an endocervical component (Elias *et al.*, 1983; Laverty *et al.*, 1989; Mauney & Sotham, 1990; Vooijs *et al.*, 1986). As a consequence, many laboratories devoted considerable time and resources to strategies aimed at increasing the proportion of smears with an endocervical component.

In Australia it became routine for laboratories to provide practitioners with sampling instruments which were specifically designed to facilitate collecting an endocervical sample. Practitioners were informed there were two reasons for the change. First, a higher detection rate of abnormalities was expected, hopefully reducing the false negative rate of screening. Second, improved detection of the precursors to adenocarcinoma was anticipated.

Victorian Cytology Service is the largest cytology laboratory in Australia, reporting in excess of 250,000 Papanicolaou smears per year. Endocervical status has been routinely reported on all smears since April 1987. During 1987 and 1988, an endocervical component was reported as being present if either columnar or squamous metaplastic cells were identified. In January 1989 more stringent criteria were introduced requiring the identification of ten or more endocervical cells singly and/or in small groups, or six or more endocervical cells in a sheet for an endocervical component to be reported as present.

Cytobrushes and cervix samplers have been routinely supplied since December 1989. Practitioners received written instructions that a cytobrush and spatula should be used together for women of any age group (except pregnant women) and that cervex samplers were a satisfactory single sampling instrument for premenopausal women. In addition, all practitioners received regular information sheets stressing the importance of the cell sample and providing an illustrated stey-by-step guide to optimal sampling techniques. A physician was employed to personally visit those practices which sought additional help. Visits targeted to practices with low success rates in sampling endocervical cells were made during 1990 and 1991.

This paper details our experience between 1987 and 1991. The proportion of smears reported as including an endocervical component has been correlated with the detection rate of high-grade intraepithelial lesions and with the reporting of abnormalities of the endocervix.

Correspondence: H. Mitchell.
Received 22 June 1992; and in revised form 17 August 1992.

### Methods

The proportion of smears reported as including an endocervical component for each of the calendar years between 1987 and 1991 was determined from computerised records. Smears which were reported as technically unsatisfactory (e.g. due to inadequate fixation, heavy inflammatory infiltrate etc) or were taken post-hysterectomy have been excluded.

The cytological reporting rate for high grade intraepithelial lesions (moderate dyskaryosis, moderate/severe dyskaryosis, severe dyskaryosis) of either squamous or adeno type was determined for each year and stratified according to endocervical status. As many women who receive these high grade reports have repeated tests as part of their further investigations, this analysis was restricted to the first reported abnormality in each year for each woman.

The rate of cytological reporting of endocervical dyskaryosis, adenocarcinoma *in situ* and adenocarcinoma of the cervix per 10,000 smears was calculated for the two time periods, 1987-1989 and 1990-1991. These time groupings correspond to periods where substantially different proportions of all smears were reported as including an endocervical component.

All analyses of abnormality rates in this study were confined to smears collected by general medical practitioners and nurse practitioners and therefore represent disease rates applicable to the general community. Smears collected by gynaecologists were excluded for two related reasons. First, in Australia smears collected by gynaecologists are frequently taken in the context of gynaecological symptoms rather than for screening purposes. (Women require a letter of referral from a general practitioner to be able to claim the cost of a visit to a gynaecologist from Medicare.) Artificial fluctuations in abnormality rates can therefore occur depending on whether or not gynaecologists routinely take smears from women with symptoms and signs consistent with malignancy. Second, during the period of this study the number of gynaecologists whose smears were reported by the Victorian Cytology Service declined as a result of a rapid expansion of private pathology laboratories. Exclusion of smears collected by gynaecologists was felt necessary to remove these extraneous influences on abnormality rates.

### Results

The proportion of smears in each year from 1987 to 1991 which were reported as including an endocervical component is shown in Table I. While some fluctuation is evident, the proportion has increased from around one half to more than three quarters, with the major increase occurring between 1989 and 1990. This time period coincided with the introduction of special sampling devices and the educational initiatives for practitioners.

The rate of reporting of high grade abnormalities per 10,000 smears in each of the calendar years is shown in Table II. With the exception of 1988, the rates are fairly stable. In particular, there is no evidence of a direct increase during 1990–1991 when a higher proportion of smears were reported as including an endocervical component. Among smears with an endocervical component, there was a decrease in the rate of reporting of each degree of high-grade intraepithelial abnormality between 1987–1989 and 1990-1991 (See Table IIIa). With the exception of CIN 3, no clear trend was apparent in the abnormality rates among the group of smears reported as not including an endocervical component (See Table IIIb).

The ratio of high-grade abnormality rates among smears with and without an endocervical component declined throughout the period of this study from 5.9 (77.8/13.2) in 1987 to 2.4 (55.2/23.3) in 1991.

**Table I** Proportion of smears reported as including an endocervical component by calendar year

| *Year* | *Proportion* |
|---|---|
| 1987[a] | 57% |
| 1988 | 53% |
| 1989 | 49% |
| 1990 | 76% |
| 1991 | 78% |

[a]Based on April–December only.

The rate of cytological reporting of adenocarcinoma of the cervix and its precursors increased from 0.66 per 10,000 smears during 1987–1989 (95% confidence interval 0.46–0.86) to 1.06 per 10,000 smears during 1990–1991 (95% confidence interval 0.76–1.37). This increase was not statistically significant.

**Table II** Rate of cytological reporting of high-grade intraepithelial abnormality per 10,000 smears by year

| | *Rate per 10,000 smears* | | | |
|---|---|---|---|---|
| *Year* | *Severe, moderate/severe, moderate dyskaryosis (95% confidence interval)* | *Severe dyskaryosis* | *Moderate/severe dyskaryosis* | *Moderate dyskaryosis* |
| 1987[a] | 46.1 (42.8–49.4) | 12.5 | 12.3 | 21.4 |
| 1988 | 71.7 (68.0–75.3) | 19.1 | 21.5 | 31.1 |
| 1989 | 48.3 (45.2–51.3) | 14.3 | 15.0 | 19.0 |
| 1990 | 50.2 (47.2–53.1) | 15.3 | 15.2 | 19.7 |
| 1991 | 49.0 (46.1–52.0) | 15.3 | 13.6 | 20.1 |

[a]Based on April–December only.

**Table III** Rate of cytological reporting of high-grade intraepithelial abnormality per 10,000 smears by year

(**a**) Among smears which were reported as including an endocervical component

| | *Severe, moderate/severe, moderate dyskaryosis* | *Severe dyskaryosis* | *Moderate/severe dyskaryosis* | *Moderate dyskaryosis* |
|---|---|---|---|---|
| 1987[a] | 77.8 | 21.8 | 21.7 | 34.4 |
| 1988 | 102.6 | 27.9 | 31.3 | 43.4 |
| 1989 | 68.6 | 21.1 | 21.2 | 26.3 |
| 1990 | 56.5 | 17.3 | 16.9 | 22.3 |
| 1991 | 55.2 | 17.4 | 15.5 | 22.4 |

(**b**) Among smears which were reported as not including an endocervical component

| | *Severe, moderate/severe, moderate dyskaryosis* | *Severe dyskaryosis* | *Moderate/severe dyskaryosis* | *Moderate dyskaryosis* |
|---|---|---|---|---|
| 1987[a] | 13.2 | 2.5 | 2.2 | 8.4 |
| 1988 | 24.7 | 5.9 | 6.9 | 11.9 |
| 1989 | 22.5 | 5.7 | 7.3 | 9.4 |
| 1990 | 23.2 | 7.0 | 7.0 | 9.3 |
| 1991 | 23.3 | 7.0 | 6.1 | 10.2 |

[a]Based on April–December only.

**Table IV** Number and proportion of all smears reported as unsatisfactory for assessment, 1987–1991

| | *Number of smears received* | *Number (%) reported as unsatisfactory* | *Number (%) with insufficient squamous cells* |
|---|---|---|---|
| 1987 | 262,721 | 1170 (0.45%) | 682 (0.26%) |
| 1988 | 252,950 | 1446 (0.57%) | 843 (0.33%) |
| 1989 | 238,164 | 1091 (0.46%) | 624 (0.26%) |
| 1990 | 255,836 | 883 (0.35%) | 538 (0.21%) |
| 1991 | 256,419 | 771 (0.30%) | 463 (0.18%) |

### Discussion

The observation from cross-sectional studies that a higher abnormality rate is reported among smears which include an endocervical component than among smears which lack an endocervical component has two possible explanations. The presence of an endocervical component could be either a marker of a group of women who are at higher risk of abnormality (possibly due to an exposed transformation zone or to reduced cell adhesiveness in the presence of disease) or, alternatively, the presence of an endocervical component could be a marker for a high quality smear such that a more comprehensive detection of abnormalities occurred.

This study, based on an analysis of more than one million Papanicolaou smears reported over 5 years, has shown that despite a very substantial increase in the proportion of smears with an endocervical component, no commensurate increase in the reporting of high-grade intraepithelial lesions of the cervix occurred. The declining ratio of reported abnormalities in smears with and without an endocervical component indicates a weakening of the relationship between endocervical status and the probability of an abnormality being reported. These findings suggests that the more likely explanation of the association between endocervical component and higher abnormality rate was that a relatively easily sampled endocervical component was a marker of women who were at higher risk of abnormality.

A number of randomised trials have recently been conducted evaluating different sampling instruments with the outcome measure of interest being the rate of cytological reporting of dyskaryosis. Four randomised trials (Goorney *et al.*, 1989; Buxton *et al.*, 1990; Selvaggi & Malviya, 1991; Szarewski *et al.*, 1990) have demonstrated an increase in the proportion of smears with an endocervical component but no commensurate increase in the detection of intraepithelial abnormalities. Wolfendale *et al.* (1987) showed an increase in the sampling of endocervical material and, while a increase in the crude rate of reporting of dyskaryotic smears was noted, when account was taken of the design of the study, no statistically significant increase in the detection of abnormalities was found. Overall the findings from these randomised trials support the findings of this current study; that is, that there may not be a commensurate increase in the reporting of intraepithelial abnormalities despite an increase in the endocervical sampling rate. Conclusions by Boon *et al.* (1989) about higher detection rates of intraepithelial neoplasia with different sampling instruments have been considered invalid by Sasieni (1991, 1992) because of flawed statistical analyses.

Over the time period of this study, the age of the women being screened by VCS did not alter to any appreciable extent; 82%–84% of smears in each year were received from women under 50 years of age. Similarly there is no evidence that the failure to show an increased reporting of high grade intraepithelial abnormalities was due to a decline in the quality of the sample of the anatomical ectocervix. Table IV shows the proportion of all smears which were reported as unsatisfactory for assessment in each of the calendar years of this study plus gives details of the number of smears which were reported as being unsatisfactory because of insufficient squamous cells. The data show that the proportion of smears reported as being unsatisfactory has declined over the period of this study.

The introduction of the new sampling instruments and the educational program for practitioners was not without cost, both in financial terms and in the use of human resources. Substantial efforts were needed to inform practitioners about how to collect a Papanicolaou smear using two instruments without suffering a deterioration in the quality of the specimen; cervical cells deteriorate rapidly after collection and there was a need to ensure that the fixation of both specimens was adequate. Many practitioners wished to use two glass slides, one for each specimen. This was considered highly undesirable as it would result in a doubling of the number of slides to be processed.

From the laboratory's viewpoint, the new policy had a number of effects. An internal retraining program was necessary for cytologists as they were unfamiliar with the full range of appearances of endocervical samples obtained using a brush. Even the requirement that all specimens have their endocervical status reported was associated with a slowing of work throughput. Perhaps more intangibly there was a disruption to the general level of confidence among the cytologists which was particularly apparent during 1988. The fact that other laboratories were reporting very much high proportions of all smears as including an endocervical component raised concerns about the false negative rate among our numerically large group of smears which lacked an endocervical component. In addition VCS cytologists were aware that other laboratories were reporting up to 15% of their smears as showing evidence of human papillomavirus effect; the comparable figure within VCS was approximately 4%. These concerns resulted in a change in reporting practice whereby more minor changes were reported as abnormal which would previously have been regarded as being within normal limits. These uncertainties probably account for the statistics for 1988 being noticeably different to the general trend. By 1989 the laboratory had, to a large extent, resumed its more long-standing profile of reporting (Mitchell & Medley, 1990).

Clearly the two reasons for advocating the change in policy to practitioners have not been fulfilled. The increase in the reporting of endocervical abnormalities may be clinically important but does not yet reach statistical significance. Adenocarcinoma of the cervix is a rare disease among the premenopausal age group which comprises the majority of participants in the screening program in Australia (Free *et al.*, 1991). We have previously shown that the sensitivity of cervical cytology for the detection of adenocarcinoma is less than for squamous carcinoma (Mitchell *et al.*, 1988). A worthwhile benefit of the changed policies will be if the accuracy of predicting disease of the endocervix improves, particularly the detection of endocervical dyskaryosis and adenocarcinoma *in situ*. Continued monitoring in these areas is occurring.

### References

BOON, M.E., DE GRAAFF GUILLOUD, J.C. & RIETVELD, W.J. (1989). Analysis of five sampling methods for the preparation of cervical smears. *Acta Cytol.*, **33**, 843–848.

BUXTON, J., LUESLEY, D., WOODMAN, C., REDMAN, C. & WILLIAMS, D. (1990). Endocervical sampling with a cytobrush does not improve cervical cytology. *J. Exp. Clin Cancer Res. (Suppl.)*, **9**, FC/78.

ELIAS, A., LINTHORST, G., BEKKER, B. & VOOIJS, P.G. (1983). The significance of endocervical cells in the diagnosis of cervical epithelial changes. *Acta Cytol.*, **27**, 225–229.

FREE, K., ROBERTS, S., BOURNE, R., DICKIE, G., WARD, B., WRIGHT, G. & HILL, B. (1991). Cancer of the cervix – old and young, now and then. *Gyn. Oncol.*, **43**, 129–136.

GOORNEY, B.P., LACEY, C.J.N. & SUTTON, J. (1989). Ayre v Aylesbury cervical spatulas. *Genitourin. Med.*, **65**, 161–162.

LAVERTY, C.R., FARNSWORTH, A., THURLOE, J.K. & BOWDITCH, R.C. (1989). The importance of the cell sample in cervical cytology: a controlled trial of a new sampling device. *Med. J. Aust.*, **150**, 432–436.

MAUNEY, M. & SOTHAM, J. (1990). Rates of condyloma and dysplasia in Papanicolaou smears with and without endocervical cells. *Diagn. Cytopath.*, **6**, 18–21.

MITCHELL, H., MEDLEY, G. & DRAKE, M. (1988). Quality control measures for cervical cytology laboratories. *Acta Cytol.*, **32**, 288–292.

MITCHELL, H. & MEDLEY, G. (1990). Age and time trends in the prevalence of cervical intraepithelial neoplasia on Papanicolaou smear tests, 1970–1988. *Med. J. Aust.*, **152**, 252–255.

SASIENI, P. (1991). Cervical samplers. *Brit. Med. J.*, **303**, 313–314.

SASIENI, P. (1992). Sampling methods for cervical smears. *Acta Cytol.*, **36**, 452–453.

SELVAGGI, S.M. & MALVIYA. (1991). Sampling accuracy of the modified Ayre spatula/Zelsmyr Cytobrush versus the modified Ayre spatula/bulb aspirator in the collection of cells from the uterine cervix. *Diagn. Cytopath.*, **7**, 318–322.

SZAREWSKI, A., CUZICK, J., NAYAGAM, M. & THIN, R.N. (1990). A comparison of four cytological sampling techniques in a genitourinary medicine clinic. *Genitourin. Med.*, **66**, 439–443.

VOOIJS, G.P., ELIAS, A., VAN DER GRAFF, Y. & POELEN-VAN DE BERG, M. (1986). The influence of sample takers on the cellular composition of cervical smears. *Acta Cytol.*, **30**, 251–257.

WOLFENDALE, M.R., HOWE-GUEST, R., USHERWOOD, M. & DRAPER, G.J. (1987). Controlled trial of a new cervical spatula. *Br. Med. J.*, **294**, 33–35.